# Long-Term Interactions of *Salmonella* Enteritidis With a Lytic Phage for 21 Days in High Nutrients Media

**DOI:** 10.3389/fcimb.2022.897171

**Published:** 2022-05-30

**Authors:** Rocio Barron-Montenegro, Dácil Rivera, María Jesus Serrano, Rodrigo García, Diana M. Álvarez, Julio Benavides, Fernanda Arredondo, Francisca P. Álvarez, Roberto Bastías, Soledad Ruiz, Christopher Hamilton-West, Eduardo Castro-Nallar, Andrea I. Moreno-Switt

**Affiliations:** ^1^ Escuela de Medicina Veterinaria, Facultad de Agronomía e Ingeniería Forestal, Facultad de Ciencias Biológicas, Facultad de Medicina, Pontificia Universidad Católica de Chile, Santiago, Chile; ^2^ Escuela de Medicina Veterinaria, Universidad Andres Bello, Santiago, Chile; ^3^ Departamento de Medicina Preventiva, Facultad de Ciencias Veterinarias y Pecuarias, Universidad de Chile, Santiago, Chile; ^4^ Instituto de Biología, Pontificia Universidad Católica de Valparaíso, Valparaíso, Chile; ^5^ Department of Biology, Emory University, Atlanta, GA, United States; ^6^ Facultad de Ciencias de la Vida, Universidad Andres Bello, Santiago, Chile; ^7^ MIVEGEC, MIVEGEC, IRD, CNRS, Université de Montpellier, Montpellier, France; ^8^ Centro de Bioinformática y Biología Integrativa, Universidad Andres Bello, Santiago, Chile; ^9^ Núcleo de Investigaciones Aplicadas en Ciencias Veterinarias y Agronómicas, Facultad de Medicina Veterinaria y Agronomía, Universidad de Las Américas, Santiago, Chile; ^10^ Instituto de Investigaciones Interdisciplinarias, Universidad de Talca, Talca, Chile; ^11^ Departamento de Microbiología, Facultad de Ciencias de la Salud, Universidad de Talca, Talca, Chile

**Keywords:** *Salmonella* Enteritidis, bacteriophage, coevolution, interaction bacteria-phage, *Salmonella* phages

## Abstract

*Salmonella* spp. is a relevant foodborne pathogen with worldwide distribution. To mitigate *Salmonella* infections, bacteriophages represent an alternative to antimicrobials and chemicals in food animals and food in general. Bacteriophages (phages) are viruses that infect bacteria, which interact constantly with their host. Importantly, the study of these interactions is crucial for the use of phages as a mitigation strategy. In this study, experimental coevolution of *Salmonella* Enteritidis (*S*. Enteritidis) and a lytic phage was conducted in tryptic soy broth for 21 days. Transfer to fresh media was conducted daily and every 24 hours, 2 mL of the sample was collected to quantify *Salmonella* OD_600_ and phage titter. Additionally, time-shift experiments were conducted on 20 colonies selected on days 1, 12, and 21 to evaluate the evolution of resistance to past (day 1), present (day 12), and future (day 21) phage populations. The behavior of the dynamics was modeled and simulated with mathematical mass-action models. Bacteria and phage from days 1 and 21 were sequenced to determine the emergence of mutations. We found that *S*. Enteritidis grew for 21 days in the presence and absence of the phage and developed resistance to the phage from day 1. Also, the phage was also able to survive in the media for 21 days, however, the phage titer decreased in approx. 3 logs PFU/mL. The stability of the lytic phage population was consistent with the leaky resistance model. The time-shift experiments showed resistance to phages from day 1 of at least 85% to the past, present, and future phages. Sequencing of *S*. Enteritidis showed mutations in genes involved in lipopolysaccharide biosynthesis genes *rfbP* and *rfbN* at day 21. The phage showed mutations in the tail phage proteins responsible for recognizing the cell surface receptors. These results suggest that interactions between bacteria and phage in a rich resource media generate a rapid resistance to the infective phage but a fraction of the population remains susceptible. Interactions between *Salmonella* and lytic phages are an important component for the rational use of phages to control this important foodborne pathogen.

## 1 Introduction

*Salmonella* spp. is a relevant zoonotic pathogen transmitted to humans through food and contact with animals (Hoelzer et al., 2011; Ferrari et al., 2019; Peruzy et al., 2020). *Salmonella* is also the most prevalent foodborne pathogen involved in outbreaks, hospitalizations, and deaths (Scallan et–al., 2011; Havelaar et al., 2015; Centers for Disease Control and Prevention, 2018). Worldwide, different food types have been associated with salmonellosis outbreaks including chicken and pork meat (Yang et al., 2010; Sasaki et al., 2012; Voss-Rech et al., 2015), seafood (Lo et al., 2017), dairy products (Van Kessel et al., 2013; da Cunha-Neto et al., 2020), nuts (Zhang et al., 2017), cereals (Davies and Wales, 2013), leafy greens (Reddy et al., 2016; de Oliveira Elias et al., 2019), and fresh fruits (Pui et al., 2011; Verma et al., 2018). While numerous interventions to reduce *Salmonella* contamination have been used, the number of human cases has not considerably decreased during the last decade (Havelaar et al., 2015; Sarno et al., 2021). Consequently, the development of new mitigation strategies to control *Salmonella* of common serotypes as *S.* Typhimurium and *S.* Enteritidis in food production is crucial.

The development of bacteriophage-based interventions is raising global attention (Ssekatawa et al., 2021). Bacteriophages (phages) are viruses that infect bacteria and cause their lysis (Kortright et al., 2019). Currently, there are commercial products available, along with reports on the use of phage as an intervention to reduce *Salmonella* contamination in different food types including sprouts seeds (Jianxiong et al., 2010), chicken meat (Augustine and Bhat, 2015; Sukumaran et al., 2015), milk (Modi et al., 2001), mixed seafood, ground beef trim (Yeh et al., 2018), and ready to eat foods (Guenther et al., 2012). Additionally, according to the NCBI database, there are more than one hundred phage genomes isolated from *Salmonella* strains^1^. There are several options for phage applications, at pre- and post-harvest showing 1-3 log bacterial reductions (Islam et al., 2021), which have increased the interest in using phages as mitigation strategy to reduce *Salmonella*. However, because phages are evolving antimicrobials, the design of rational phage-based mitigation strategies in food requires understanding the coevolutionary dynamics of bacteria and lytic phages under different conditions (Bohannan and Lenski, 2000; Koskella and Brockhurst, 2014).

Bacteria-phage interactions produce reciprocal coevolution in the bacterial host and the lytic phage. These interactions occur in all the steps of the phage infection cycle (Labrie et al., 2010). The initial interaction is the adsorption, in which phage tails recognize and attach to the surface receptors of bacteria such as lipopolysaccharides (LPS) and/or surface proteins (as flagella and outer membrane proteins) (Kortright et al., 2019). Bacteria are then capable to develop resistance mechanisms such as modified or blocked receptors (Moller et al., 2019), or the acquisition of spacers in the CRISPR-Cas systems, which have been described in *Salmonella* (Barrangou et–al., 2007; Medina-Aparicio et al., 2018). The bacterial resistance mechanisms to phages are crucial for their survival of phage lysis, while phages coevolved and contra-adapt to phage resistance (Borin et al., 2021).

The most reported dynamics for bacteria-phage interactions is the antagonistic coevolution that falls into two different categories, i) arms race dynamics where new alleles allow bacterial resistance and phage infectivity over time, in which bacteria become more resistant to phages from the past than to present and future phages (Brockhurst et al., 2014); and ii) fluctuating selection dynamics, in which phage evolve to infect common bacterial genotypes, given an advantage to infrequent genotypes that increase in rate (Woolhouse et al., 2002; Avrani et al., 2012). To date, the majority of the experimental coevolution studies have focused on a limited number of bacterial models (*E. coli* and *Pseudomonas fluorescens* SBW25) (Brockhurst et al., 2007). To our knowledge, only one study exploring *Salmonella* and lytic phages interactions has been published (Holguín et al., 2019). Therefore, it is important to improve our understanding of *Salmonella* and phage interactions in different conditions.

In the present study, we performed a coevolution assay for *Salmonella* Enteritidis and a lytic phage in a high level of nutrients media. To assess the effect of the interactions between *S*. Enteritidis and the phage; we studied the abundance of *S*. Enteritidis and the phage, modelled their interactions, characterized the evolutionary model, and identified genomic changes in *S*. Enteritidis and the phage upon 21 days of coevolution.

## 2 Materials and Methods

### 2.1 *S*. Enteritidis and Phage vB_Sen_STGO-35-1 Isolates and Culture Conditions

#### 2.2.1 Conditions of Culture Bacteria and Phage

*Salmonella* Enteritidis strain DR016 was isolated from chicken feces in backyard farms of central Chile (Rivera et al., 2018). This isolate is sequenced and deposited under CFSAN035147 accession number. *S*. Enteritidis was used as a host for the coevolution experiment in trypticase soy broth (TSB, Bection-Dickinson, Franklin Lakes, NJ, USA). *Salmonella* was grown in TSB for 16 h at 37°C and stored with 20% of glycerol at -80°C. Phage vB_Sen_STGO-35-1 (STGO-35-1) was isolated from a backyard chicken flock, using a strain of *S*. Enteritidis as host, according with the protocol described by Rivera et al. (2018). A complete description of the used phage, annotation, and growth conditions is in Rivera et al. (2022). High titer phage stock was stored at 4°C in SM (50 mM Tris-Cl, pH 7.5; 0.1 M NaCl; and 0.01 mM MgSO4) buffer.

### 2.2 Coevolution-Assay

**Figure 1:**
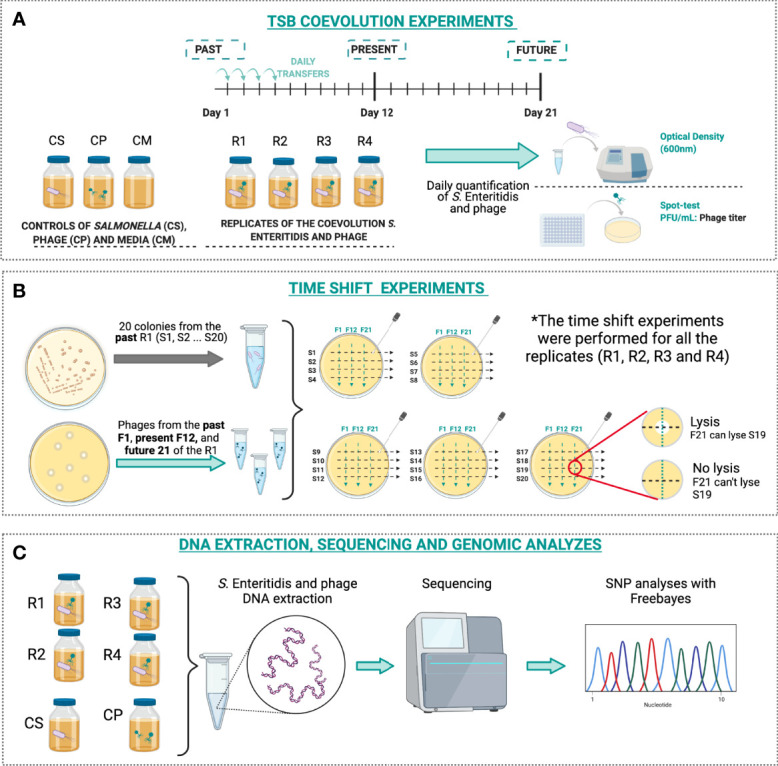
Schematic illustration of the experimental design. **(A)** Experimental design for the coevolution dynamics. Four replicates were placed (R1, R2, R3, and R4) for the infection of *S*. Enteritidis with the phage. Daily transfers were performed to fresh media, and samples were analyzed for viral and bacterial quantification. **(B)** Time shift experiment for past, present, and future phage and bacterial populations, for this, 20 colonies were selected for *S*. Enteritidis in all the three times for the four replicates and were tested against phages from the past, present, and future. * indicates the replicates in which the time shifts were performed. **(C)** The extraction of the DNA of *S*. Enteritidis was conducted for *S*. Enteritidis of days 1 and 21 for all replicates and for phage from R1 at days 1 and 21. The SNP analyzes were performed with Freebayes using the CS as the original *S*. Enteritidis.

The coevolution experiment one-host-one-phage was conducted using *S*. Enteritidis DR016 and phage STGO-35-1 at MOI 0.01 (Buckling and Rainey, 2002). The bacteria and the phage were cultured in flasks with 9 mL TSB and incubated at 37°C with 100 rpm of shaking for 21 days. Daily transfers were performed with 90 µL of the cultures into 9 mL of fresh TSB for revitalization (Barbosa et al., 2013). Four replicates were conducted, along with three controls, including one flask with only *S.* Enteritidis, one with only STGO-35-1 phage, and one without inoculation **(** **Figure 1A** **).** Daily samples were collected for bacterial and viral quantification from all the replicates and controls. Samples from days 1, 12, and, 21 were centrifugated at 14,000 rpm for 10 min and the supernatants were filtered through 0.2 µm to store coevolved phage particles; the pellet was resuspended in TSB and frozen in 20% glycerol at -80°C to store coevolved *Salmonella* for sequencing (see below).

### 2.3 Estimation of *S*. Enteritidis and Phage Abundance

During the experiment, daily samples of 2 mL were collected in each replicate. The estimation of *S*. Enteritidis was measured with a spectrophotometer using OD_600nm_ (C40, Implen-ALE, Munich, Germany). The phage titer was determined using unevolved *S*. Enteritidis and titer was conducted using the “spot-test”, as previously described (Rivera et al., 2018) **(** **Figure 1A** **).** We tested whether phage or bacteria population were affected by time and the bacteria or phage presence, respectively. The data was analyzed using a generalized linear mixed model (GLMM) with binomial errors and the logit- link function. Replicates were included as a random effect and both bacterial or phage presence and time sample were included as fixed factors. The analysis was conducted using RStudio statistical software, version 3.5.2. Model was run with the ‘glmer’ function in the ‘lme4’ package in R.

### 2.4 *S*. Enteritidis Resistance and Phage Infectivity Evaluated by Time-Shift

To evaluate bacterial resistance and phage infectivity, generated through the coevolution experiment, phage and *S*. Enteritidis populations were sampled at three different times, characterized as past (day 1), present (day 12), and future (day 21). For the phage, 1 mL of lysate was stored as described above. For *S*. Enteritidis, cultures were serially diluted to 1·10^6^ CFU/mL, a total of 100 µL were streaked on TSA plates and 20 colonies were randomly selected for purification and frozen with 20% glycerol at -80°C. The proportions of *S*. Enteritidis resistance to phage and phage infectivity in *S*. Enteritidis was calculated from the three times by cross streaking the isolated 20 colonies across lines crossed by streaked phages, as previously described (Koskella and Brockhurst, 2014) **(** **Figure 1B** **)**. Data were scored as 0 for no lysis observed and 1 when lysis was observed. To assess resistance and infectivity over time and differences between replicates, we first examined variation in phage infectivity using a generalized linear mixed model (GLMM) with binomial errors and the logit- link function to test whether phage local adaptation was affected by the time sample in which bacteria was collected using RStudio (version 3.5.2). In this analysis, replicates were included as a random effect and both bacterial and phage time samples were included as fixed factors. As the replicates had a similar behavior, we used a generalized linear mixed model (GLMM) to examine variation in bacterial resistance where replicates were included as a random effect and both bacterial and phage time samples were included as fixed factors.

### 2.5 Mathematical Model for Population Dynamics

A population model adapted from Chaudhry et al. (2018) was used to estimate changes in populations of bacteria and phage over time. This model consists of a system of four differential equations for resource (R), susceptible cells (N), resistant cells (NR), a virulent phage (PV) and a Monod function (Equations 1-4, Chaudhry et al., 2018) (**Table 1**). The equations were programmed and solved in the software Berkeley Madonna 10.2.8 with the Euler method and a DT value of 1·10^-4^. For serial-passage simulations, a mathematical term was added to each equation to achieve the dilution of the populations proportional to the dilution factor used in the time-shift experiments for every 24 hours of integration.

**Table 1:** Simulation parameters used in the mathematical model.

Parameter	Values (dimensions)	Description	Source
*v, v_r_*	1 (*h* ^-1^)	Maximum growth rates	Rivera et al., 2022
*µ_R_, µ_N_*	5*e* ^-6^, 5*e* ^-5^ (*h* ^-1^)	Transitions N_r_ ➔ N, N ➔ N_r_	Chaudhry et al. (2018)
*β*	60 (*PFU* · *CFU* ^-1^)	Burst size	Rivera et al., 2022
*K*	1	Monod constant	Stewart and Levin (1973)
*e*	5*e* ^-7^ (*µg* · *CFU* ^-1^)	Conversion efficiency	Stewart and Levin (1973)
*δ*	1*e* ^-8^ (*h* ^-1^ · *mL^-^* ^1^)	Adsorption rate	Rivera et al., 2022

These parameters are specific for phage STGO35-1 as the burst size, the adsorption rate, and for S. Enteritidis the parameter of the maximum growth rate.

### 2.6 Sequencing of *S*. Enteritidis and Phage vB_Sen_STGO-35-1

Sequenced DNA of populations of *Salmonella* Enteritidis corresponded to i) a reference genome represented by *S.* Enteritidis before the coevolution assays, ii) *S.* Enteritidis coevolved from day 1 from all 4 replicates, iii) *S.* Enteritidis coevolved from day 21 from all 4 replicates, and iv) controls (only *Salmonella* and only phage) from days 1 and 21 (**Figure 1C**). The DNeasy Blood and Tissue Kit (Qiagen, Valencia, CA, USA) was used to purified DNA from overnight cultures. DNA concentration and quality were measured by calculating the optical density ratio 260/280 with a MaestroNano Pro Spectrophotometer (Maestrogen Inc., Hsinchu, Taiwan). The genomic DNA libraries were prepared using the Nextera XT library preparation kit (Illumina, San Diego, CA, USA), and paired-end sequencing were conducted using HiSeq technology from Illumina in Novogen Technology Co., Ltd, in the United States. Sequences were analyzed with FastQC (Babraham Bioinformatics, 2016) and then were quality trimmed using Trimmomatic (Bolger et al., 2014), trimming at an average Q score below 25 and with a minimum length of 50bp. Trimmed Illumina reads from *S*. Enteritidis before coevolution (reference genome) were assembled using the ‘isolate’ mode in SPAdes v3.14.0. Then, scaffolds with length <1kb were removed from the assembly. Blasting resulting scaffolds against an NCBI database of *Salmonella enterica*, showed that all scaffolds presented >95% of identity. One scaffold with 100% identity to *Salmonella enterica* plasmid pSJTUF10978 (CP015525) was removed. The final assembly had a consensus length of 4,647,970 bp spanning 24 scaffolds, with an N50 value of 490,728 bp. The average genome coverage was 134X with a GC % of 52.1%. Finally, collinearity with *S. enterica* (CP050716) was evaluated using D-GENIES (Cabanettes and Klopp, 2018) showing that the majority of the *S. enterica* genome (98.79%) was covered by the assembly. Gene annotation of this assembly was performed with RASTtk (Brettin et al., 2015). The same approach was used to assemble and annotate individual genomes for each sample from day 1 and day 21.

Sequenced phage corresponded to i) reference phage represented by STGO-35-1 before the coevolution, ii) coevolved phage from day 1 from replicate 1, and iii) coevolved phage at day 21 from replicate 1. Phenol/chloroform extraction of DNA and precipitation with ethanol were performed for phage STGO-35-1, as previously described (Rivera et al., 2022). DNA concentration was determined by OD measurement with the Maestro Nano Pro-Spectrophotomether (Maestrogen Inc,. Hinschu, Taiwan) and quality was determined with the ratio of 260/280 nm. Sequencing libraries and sequencing were conducted at Novogen Technology Co., Ltd, in the United States. Filtered sequencing reads were then quality trimmed using Trimmomatic (Bolger et al., 2014), trimming at an average Q score below 25 and with a minimum length of 50bp. Trimmed Illumina reads from samples were then co-assembled using the ‘isolate’ mode in SPAdesv3.14.0. One scaffold of 47,072 bp with 21,487x of coverage was assembled. Comprehensive genome characteristics are described in Rivera et al. (2022). Sequences for bacteria and phage are available at ncbi bioproject PRJNA821546.

### 2.7 SNP Analyses for *S*. Enteritidis and Phage vB_Sen_STGO-35-1

For aligning the quality filtered reads to the assembled *Salmonella* and phage genomes, we used Bowtie2 (version 2.4.1), using default parameters (Langmead and Salzberg, 2012). Duplicate reads were removed with the ‘samtools markdup’ tool. Freebayes (version 0.9.18-1) (Garrison and Marth, 2012) was used for variant calling **(** **Figure 1C** **)**. SNPs were required to have a minimum Phred score of 20, quality of mapped read >30; more than 4 reads covering the base in every genotype and a minimum alternate allele fraction of 0.2 and at least 10 bp before and after the variant. VCF files were regularized using the vcf allelic primitives’ module of vcflib v1.0.0-rc2 (https://github.com/ekg/vcflib), which splits adjacent SNPs into individual SNPs, left-aligns indels, and regularizes the representation of complex variants. SnpEff (version 4.0e) (Cingolani et al., 2012) was used to annotate variants. The SNPs found in the reference *S*. Enteritidis were eliminated, as they were not the result of exposure of *S*. Enteritidis to the phage.

## 3 Results and Discussion

### 3.1 Dynamics of Growth of *S*. Enteritidis and a Lytic Phage for 21 Days

#### 3.1.1 Population Density of *S.* Enteritidis and vB_Sen_STGO-35-1 Phage

**Figure 2:**
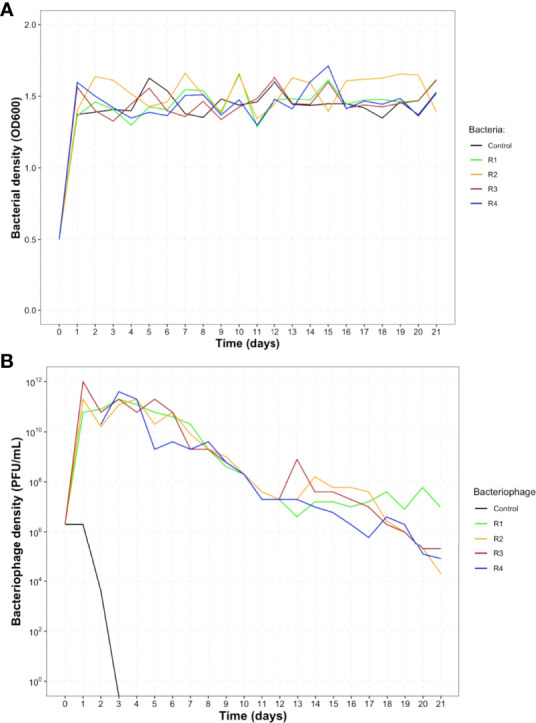
Population density of *S.* Enteritidis and a lytic phage for 21 days. **(A)** Population density of *S*. Enteritidis for the 21 days of experimental coevolution. **(B)** Phage titer for the 21 days of experimental coevolution. For both, the control without the phage/bacteria are in black, and the four replicates are identified as replicate 1 (R1) in green, replicate 2 (R2) in orange, replicate 3 (R3) in red, and replicate 4 (R4) in blue. For both charts, day 0 corresponds to the time of the infection and day 1, 24 hours later.

The quantifications of *S*. Enteritidis showed that it survived in the presence of the phage for 21 days **(** **Figure 2A** **).** In general, the four replicates behaved homogeneously, with an exponential multiplication on day one and then maintaining an optical density between 1.3 and 1.6 until day 21. In the control, where only *S*. Enteritidis was inoculated, the optical density was similar among the replicates, with no significant changes. These results suggest that the phage STGO-35-1 in the media did not affect the abundance of *S*. Enteritidis using a MOI of 0.01. We also observed a homogeneous behavior among the replicates in terms of phage populations. On day one, the phage had a higher titer and then declined steadily over time but did not go extinct. The control with only phage inoculation was indetectable by PFU quantification after day 3 **(** **Figure 2B** **).** Our results could indicate that *S*. Enteritidis of day 1 (past) developed resistance to the phage. Still, a minor population of *S*. Enteritidis remains susceptible and allows the phage to replicate for 21 days. Our results indicate that a subpopulation of *Salmonella* is still phage-susceptible in all the replicates tested. We observed that the phage titer decreased over time (10^4^ PFU/mL) (**Figure 2B**), which could be explained by resistance mechanisms acquired by *Salmonella* (see below). Similar behaviors of host-phage coexistence were reported for *Streptococcus thermophilus* and a lytic phage (Weissman et al., 2018).

**Figure 3:**
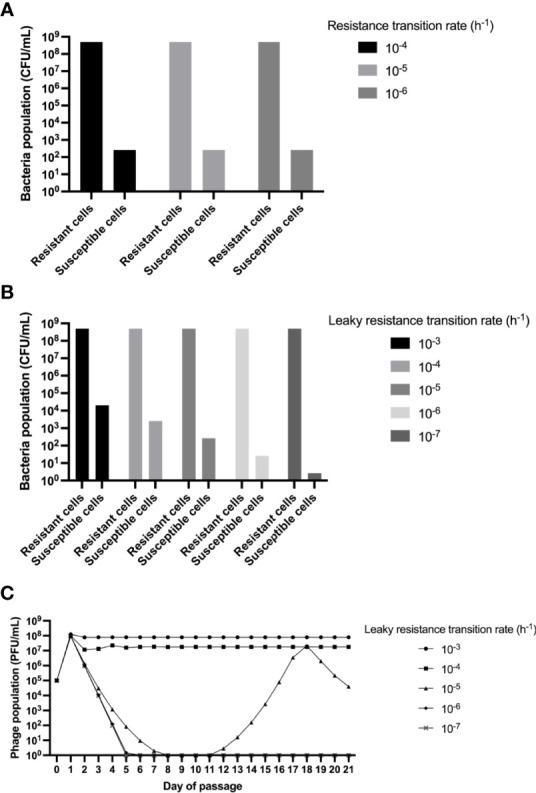
Mathematical model and simulations. Computer simulation results for the changes in the densities of phage populations. Unless otherwise stated, the parameters used to simulate were: k=1, e= 5·10^-7^ μg/cell, v 2.0 h^-1^, δ=2·10-7 h^-1^cell^-1^, β=60 phages/cell. **(A)** Changes in phage densities after 23 hours when testing different values of μ_N_. **(B)** Changes in phage densities after 23 hours when testing different values of μ_R_. **(C)** Changes in phage densities for 21 days of serial passage (dilution factor of 0.01) when testing different values of μ_R_.

Consistent with the previous results, our mathematical modelling confirms the possibility of selection of a dominant population of resistant bacteria (N_R_) at 24 hours, which explains why the phage titer decreased **(** **Figure 3A** **)**. This result is repeated when different rates for the transition of susceptible to resistant bacteria (μ_N_) are used in the simulations. Interestingly, in all the cases, a minor population of coexisting susceptible bacteria (N) is present in the culture, which could explain why the phage survive for the 21 days, even when the dominant population of *Salmonella* are resistant. The latter observation could support the hypothesis of the cohabiting population of susceptible bacteria being able to host the phage multiplication and its maintenance in the culture; however, the density of this population (1·10^2^ CFU/mL) might be too low to satisfy the probability of infection (Schrag and Mittler, 1996). Alternatively, the proportion of susceptible population could be higher than predicted in the model; furthermore, it could be transitioning or “leaking” at a higher rate than what is known for *E. coli* (Chaudhry et al., 2018). We tested this option performing simulations with different rates of leaky resistance transition (μ_R_) but maintaining the resistance generation rate constant over time. The results indicate that dominance of resistance at 24 hours occurs in most cases (**Figure 3B**). Although present, the population of susceptible bacteria is still maintained as a minority, and as expected, its density is directly proportional to the value of μ_R_.

Remarkably, when the same approach of **Figure 3B** is used in serial-passage for 21 days, we can simulate the dynamics of the virulent phage population (**Figure 3C**). In this case, even with dominance of resistance in the majority of cases, we can only achieve phage maintenance as seen in our experiments (**Figure 2B**) when the leaky resistance transition rate is equal or higher than our rate of generation of resistance, indicating that while selection of resistant mutants is made in the first 24h, it could be highly unstable, or leaky. Moreover, as stated before, leaky resistance transition rates that produce ~1·10^2^ CFU/mL or less susceptible cells are not sufficient to maintain the phage replication through the passages (**Figures 3A, B** **).** Results found here are consistent with the “leaky-model resistance”, which has been proposed as a mechanism responsible for maintaining phages in populations dominated by highly resistant bacteria (Chaundry et al., 2018). In this model, a considerable rate of genetic and/or phenotypic reversion of resistant to susceptible bacteria occurs, and then populations can find stability through low rates of multiplication (Chaundry et al., 2018). The leaky resistance model was described for *E. coli* and a virulent mutant of phage Lambda; and in *Salmonella*, a previous study that coevolved *S*. Enteritidis for 12 days with a lytic phage, did not show population density for susceptible bacteria (Holguín et al., 2019). This study tested the population density in a high nutrient media, where we observed that *S*. Enteritidis acquired resistance the first 24 hours; the foregoing justifies the need to evaluate the interactions between *Salmonella* and lytic phages under different conditions, to understand these behaviors better, and to rationally use phages as an alternative for the biocontrol of *Salmonella* in complex systems such as foods.

#### 3.1.2 Coevolutionary Dynamics of S. Enteritidis and a Lytic Phage

**Figure 4:**
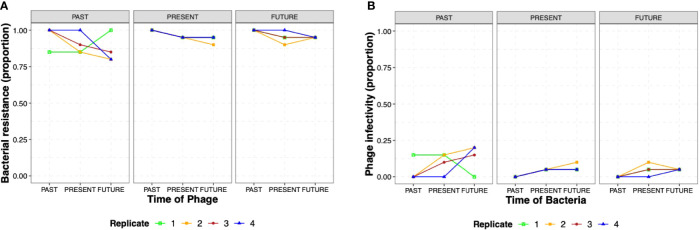
Coevolutionary dynamics of *S.* Enteritidis and a lytic phage. **(A)** Proportions of the 20 colonies of *S*. Enteritidis that presented resistance to phage populations from i) the past (day 1), ii) the present (day 12), and iii) the future (day 21) for the four replicates (R1, R2, R3 and R4). **(B)** Proportions of phage infectivity, represented by lysis on the 20 isolated *Salmonella* from i) the past (day 1), ii) the present (day 12), and iii) the future (day 21) for the four replicates (R1, R2, R3 and R4). The figure does not show several times-points (past, present, and future) of replicates because proportions overlapped among replicates. Raw data of *S*. Enteritidis resistance and phage infectivity (proportion) are shown in **Supplemental Tables 2, 3**, respectively.

To evaluate coevolutionary dynamics, we measured the proportion of phage resistance in *S*. Enteritidis from populations obtained from i) the past (day 1), ii) the present (day 12), and iii) the future (day 21); (**Figure 1B**) these against phages from the same three time points (1, 12, and 21). *S*. Enteritidis developed a high resistance proportion (0.75 – 1.00) against the phages for all the different times (1, 12, and 21 days) in all four replicates, without significant differences between the different phage times or replicates (GLMM, p> 0.05) **(** **Figure 4A** **).** We also tested phage infectivity of isolated *Salmonella* obtained from i) the past (day 1), ii) the present (day 12), and iii) the future (day 21). Conversely, phage infectivity was found in low proportions (0.00 – 0.25) in all three times and replicates as well, similarly, without significant differences between *Salmonella* time or replicates (GLMM, p> 0.05) **(** **Figure 4B** **)**. Overall, our results show a bacterial resistance mechanism emergence during the first day of coevolution that lasted through the 21 days of the experiments. Over time, high resistance to phage was reported for other bacterial genera such as *Vibrio* sp. and *E. coli* with lytic phages (Barbosa et al., 2013; Scanlan et al., 2019). But this behavior was also observed in *S.* Enteritidis in a previous study that reported the absence of evidence of antagonistic coevolution between phage ϕSan23 and *S*. Enteritidis s25pp (Holguín et al., 2019). Importantly, while this previous study used a different MOI (10 versus 0.01); both studies showed similar behaviors. Further studies testing different MOIs and phage combinations are necessary to understand if the interactions are MOI-dependent. The coevolutionary dynamics of *S*. Enteritidis and phage STGO-35-1 did not show an arm race model or antagonistic coevolution in the conditions studied here (media, temperature, MOI, days). Importantly, the interactions between bacteria and lytic phages depend on the host and the environment with controlled parameters as temperature, resources, and multiplicity of infection (Hernandez and Koskella, 2019).

Our findings indicate that *S.* Enteritidis could have an advantage over phages in this model of TSB, suggesting, for instance, that it could be easier for *Salmonella* to mutate the phage receptor than for the phage to find the exact mutation for the new receptor under these experimental conditions (Lenski and Levin, 1985). Conversely, the nutrient availability in the used model may facilitate phage resistance mechanisms (Lopez‐Pascua and Buckling, 2008) but is far from what *Salmonella* is exposed in natural conditions. The most important mechanisms that explain the increase in coevolution rates are related to the increase in productivity in the environment (i.e., nutrients, water, temperature, movement) (Hernandez and Koskella, 2019). This increase in productivity may contribute to an increase in the growth of bacterial and phage populations, which would increase the available supply of genetic variation on which selection would act (Hernandez and Koskella, 2019). Then, environmental characteristics are likely to be important when studying the emergence of phage-resistant bacteria, as was observed in the case of the DSM3 phage that infected *P. aeruginosa* (Alseth et al., 2019). Thus, future studies could explore the coevolutionary dynamics of *Salmonella* with lytic phages under environments more similar in terms of resources and other bacteria found in complex environments.

### 3.2 Identification of Single Nucleotide Polymorphisms (SNPs) in Coevolved *S*. Enteritidis and Lytic Phage

#### 3.2.1 SNPs Found in *S.* Enteritidis and in Phage vB_Sen_STGO-35-1

**Figure 5:**
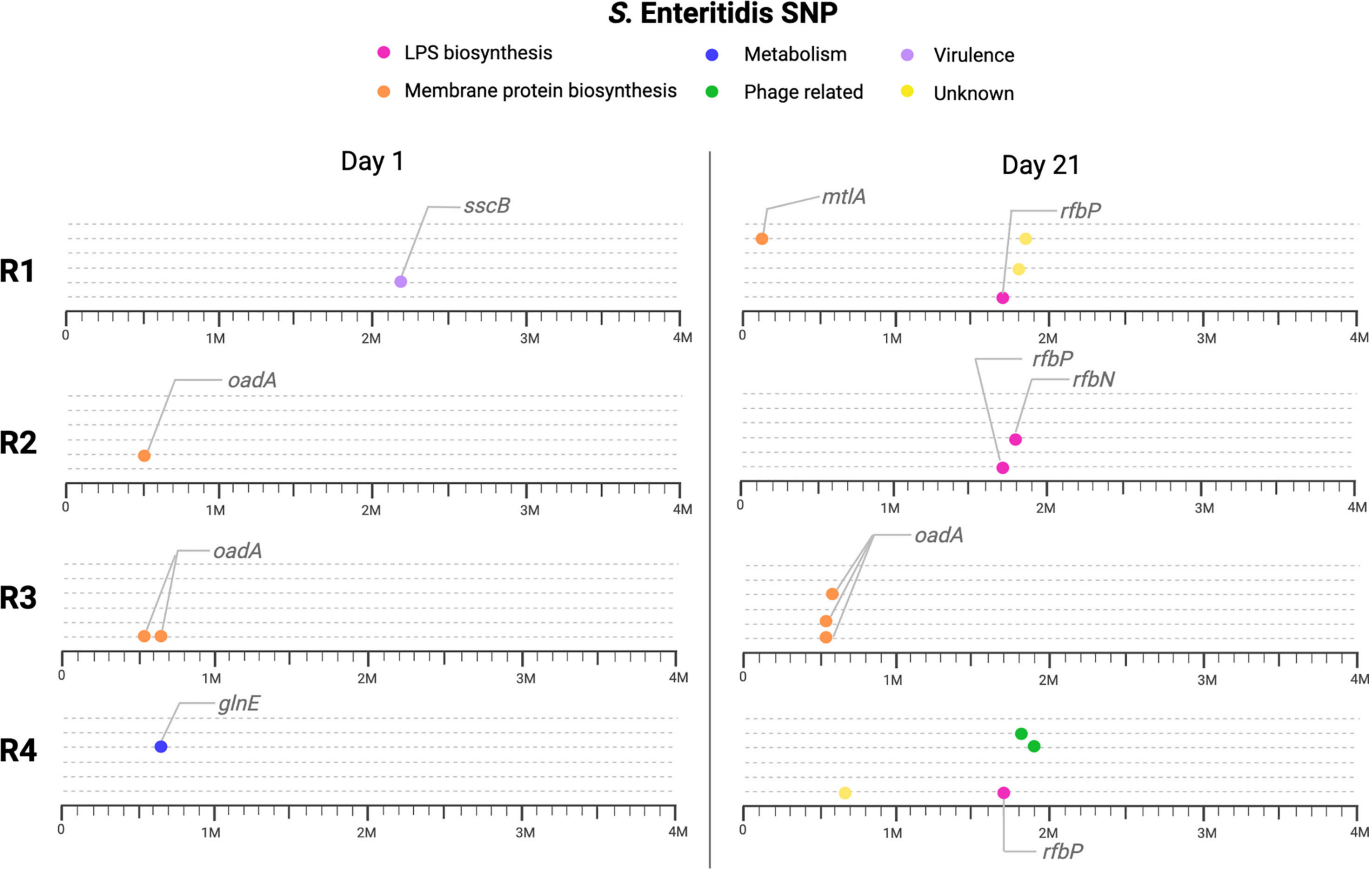
SNP found in *S*. Enteritidis for day 1 (present) and day 21 (future) for the four replicates (R1, R2, R3 and R4). Genes indicated with lines are the most relevant, we also classified the genes with SNPs in six different categories. Pinks circles indicate genes associated with LPS biosynthesis, in orange membrane protein biosynthesis.

We sequenced *S.* Enteritidis populations from days 1 and 21 in all four replicates to determine SNPs in coevolved *Salmonella* with the lytic phage. We found 31 SNPs distributed in the replicates for days 1 and 21 **(** **Figure 5** **)**. SNPs were classified into four categories according to nucleotide changes and putative impact. Specifically, in i) missense presenting a moderate impact (13 SNPs), ii) frameshift presenting a high impact (3 SNPs), iii) stop gained presenting a high impact (1 SNP), and iv) intergenic mutations presenting a lower impact (9 SNPs). At day one, 6 SNPs were identified, most of them being missense (5 SNPs), followed by intergenic (1 SNP). These SNPs, were found in genes encoding membrane proteins as the oxaloacetate decarboxylase and undecaprenyl-phosphate-galactosyltransferase, involved in the synthesis of antigen-O for the LPS (Patel et al., 2012; Xu et al., 2020). Importantly, at day 21, more SNPs were found, with 20 SNPs identified, most of them in genes involved in LPS biosynthesis and membrane proteins. Notably, potential receptors on the bacterial surface which are recognized by phages include structures such as porins, portions of the lipopolysaccharide (LPS), and flagellar proteins (Bertozzi Silva et al., 2016). Our results found SNPs classified as frameshift with high impact in three replicates in the *rfbP* gene at day 21. This gene is involved in the biosynthesis of LPS antigen O, described as phage receptor in Gram-negative bacteria (Bertozzi Silva et al., 2016).

In general, identified SNPs were present in genes encoding proteins involved in different processes, such as metabolism, virulence factors, LPS biosynthesis, and membrane protein biosynthesis **(** **Figure 5** and **Supplemental Table 1** **),** though SNPs differ in the two times analyzed (day 1 and 21), suggesting that the mechanism for resistance is dynamic and can change through, and between, replicates. It would be interesting to evaluate if mutations observed at day 21 are more stable or can still be subjected to further modifications. In a previous study conducted in *S.* Enteritidis, most of the SNP at coevolved *Salmonella* with a lytic phage were associated with cell wall proteins and capsule proteins (Holguín et al., 2019). Other models, as published by Alseth et al. (2019), revealed that most of the SNPs found on *Pseudomonas* after exposure to DSM3 were also on membrane receptor. Additionally, different studies report that mutation in LPS genes induces phage resistance to *Salmonella* (Rivera et al., 2019; Fong et al., 2020). This indicates that the most described mechanism for phage resistance in coevolution assays represent modification of protein receptors or exopolysaccharide, similar to the results found here.

To determine SNPs in the lytic phage STGO-35-1, we sequenced phage isolated from day 1 and 21. A comprehensive report of genomic characteristics of this phage was recently reported (Rivera et al., 2022) and a short time exposure showed SNPs in receptor binding proteins. Further, in this study, we also identified two SNPs in sequenced phage from day 21, and these two SNPs were found in genes annotated as tails spike proteins, which are involved in recognizing the membrane receptors in the bacteria. The SNPs identified in the phage at day 21 were one missense mutation localized in a tail spike protein, generating an aminoacidic change from glutamic acid to glycine in the phage of the future (day 21). Another missense mutation was found in a phage tail, in which a glutamic acid was changed by a cysteine, and this amino acid had a hydrophobic nature to difference a glutamic acid, that is hydrophilic. These changes in the aminoacidic sequence of the tail proteins, responsible for recognizing the bacterial receptor, could explain why the phage survived 21 days as a counter-resistance mechanism. In addition to the leaky resistant model predicted by the mathematical modeling, both could explain the low infectivity for the tested time and conditions. These results contrasted with those results found in Holguín et al. (2019), where no significant change was described in phage genomes infecting *S*. Enteritidis. However, our results indicate a potential counter resistance mechanism in phage STGO-35-1 to overcome the bacteria resistance developed.

## 4 Conclusions

Results presented here contribute to expand our current knowledge on coevolution dynamics using a model of *Salmonella* Enteritidis and a lytic phage vB_Sen_STGO-35-1 in a rich nutrient media for 21 days. Our study highlights the potential of S. Enteritidis to co-evolve with lytic phages in a high nutrient environment by rapidly developing a resistant population but maintaining a low frequency of susceptible bacteria available to the phage for replication. The genomics data showed that *Salmonella* have different SNPs in response to the phage presence and the phage had less SNPs than the bacteria, but in important proteins described as bacterial receptors While most of the changes occurred during the first 24 hours, initial resistance mechanisms could change throughout the 21 days as observed in our sequencing results. Understanding these interactions contributes to the responsible use of the phages as biocontrol tools.

## Data Availability Statement

The datasets presented in this study can be found in online repositories. The name of the repository and accession number can be found below: NCBI; PRJNA821546.

## Author Contributions

RB-M, DR, FPÁ, and AM-S: conceived the idea, designed and conducted the experiments; MS, RG, JB, FA, SR, and CH-W: analyzed and interpreted data; RB-M, DR, DÁ, RG, and AM-S: wrote the manuscript, RB: discussed the results and critically revised the manuscript. All authors contributed to the article and approved the submitted version.

## Funding

We acknowledge the funding sources: 1 [ANID FONDECYT 1181167 to AIMS], 2 [ANID Millennium Science Initiative/ Millennium Initiative for Collaborative Research on Bacterial Resistance NCN17_081 to AM-S], and ANID FONDECYT 3210317 to DA.

## Conflict of Interest

The authors declare that the research was conducted in the absence of any commercial or financial relationships that could be construed as a potential conflict of interest.

## Publisher’s Note

All claims expressed in this article are solely those of the authors and do not necessarily represent those of their affiliated organizations, or those of the publisher, the editors and the reviewers. Any product that may be evaluated in this article, or claim that may be made by its manufacturer, is not guaranteed or endorsed by the publisher.

## Footnotes

1
http://www.ncbi.nlm.nih.gov/nuccore. Accessed on March 2022
