# Supplementary material for: Long-Term Interactions of Salmonella Enteritidis With a Lytic Phage for 21 Days in High Nutrients Media
Source: Front Cell Infect Microbiol. 2022 May 30;12:897171. doi: 10.3389/fcimb.2022.897171 (PMC9196899; doi:10.3389/fcimb.2022.897171)
Supplement: Supplementary file 1 [file DataSheet_1.docx]

**Supplemental Table 2.** Raw data of Figure 4A “Coevolutionary dynamics of *S.* Enteritidis and a lytic phage”. Media sympatric of *Salmonella* resistance in phages populations from i) the past (day 1), ii) the present (day 12), and iii) the future (day 21) for the four replicates (R1, R2, R3 and R4).

| **MEDIA SYMPATRIC *Salmonella* Enteritidis RESISTANCE** | | | | |
| --- | --- | --- | --- | --- |
| **Time *S.* Enteritidis** | **Replica** | **Phage Past** | **Phage Present** | **Phage Future** |
| **Past** | 1 | 0.85 | 0.85 | 1.00 |
| **Past** | 2 | 1.00 | 0.85 | 0.80 |
| **Past** | 3 | 1.00 | 0.90 | 0.85 |
| **Past** | 4 | 1.00 | 1.00 | 0.80 |
| **Present** | 1 | 1.00 | 0.95 | 0.95 |
| **Present** | 2 | 1.00 | 0.95 | 0.90 |
| **Present** | 3 | 1.00 | 0.95 | 0.95 |
| **Present** | 4 | 1.00 | 0.95 | 0.95 |
| **Future** | 1 | 1.00 | 0.95 | 0.95 |
| **Future** | 2 | 1.00 | 0.90 | 0.95 |
| **Future** | 3 | 1.00 | 0.95 | 0.95 |
| **Future** | 4 | 1.00 | 1.00 | 0.95 |

**Supplemental Table 3.** Raw data of Figure 4B “Coevolutionary dynamics of *S.* Enteritidis and a lytic phage”. Media sympatric of phage infectivity of isolated *Salmonella* from i) the past (day 1), ii) the present (day 12), and iii) the future (day 21) for the four replicates (R1, R2, R3 and R4).

| **MEDIA SYMPATRIC PHAGE INFECTIVITY** | | | | |
| --- | --- | --- | --- | --- |
| **Time *S.* Enteritidis** | **Replica** | **Phage Past** | **Phage Present** | **Phage Future** |
| **Past** | 1 | 0.15 | 0.15 | 0 |
| **Past** | 2 | 0 | 0.15 | 0.2 |
| **Past** | 3 | 0 | 0.1 | 0.05 |
| **Past** | 4 | 0 | 0 | 0.2 |
| **Present** | 1 | 0 | 0.05 | 0.05 |
| **Present** | 2 | 0 | 0.05 | 0.1 |
| **Present** | 3 | 0 | 0.05 | 0.05 |
| **Present** | 4 | 0 | 0.05 | 0.05 |
| **Future** | 1 | 0 | 0.05 | 0.05 |
| **Future** | 2 | 0 | 0.1 | 0.05 |
| **Future** | 3 | 0 | 0.05 | 0.05 |
| **Future** | 4 | 0 | 0 | 0.05 |
