# Supplementary material for: Long-Term Interactions of Salmonella Enteritidis With a Lytic Phage for 21 Days in High Nutrients Media
Source: Front Cell Infect Microbiol. 2022 May 30;12:897171. doi: 10.3389/fcimb.2022.897171 (PMC9196899; doi:10.3389/fcimb.2022.897171)
Supplement: Supplementary file 2 [file Table_1.docx]

| **Replicate** | ***S.* Enteritidis** | **Function** | **Gen mutated** | **Gene product activity** | **Chromosome position (bp)** | **Effect type** | **Putative impact** |
| --- | --- | --- | --- | --- | --- | --- | --- |
|  |  |  |  |  |  |  |  |
| **1** | PAST | Secretion system III | *sscB* | Its a chaperone for SseF a effector protein involve in TSIII | NODE1_947512 | Missense | MODERATE |
|  |  | Transcription | *23S* | 23s RNA its a component of large subunits 50S of ribosome | NODE22_673 | Interegenic | MODIFIER |
|  | FUTURE | Unknown | *ydfU* | Uncharacterized protein of ydfU family | NODE16_213 | Frameshift | HIGH |
|  |  |  | *-* | Hypothetical protein | NODE16_2853 | Missense | MODERATE |
|  |  | LPS byosynthesis | *rfbP* | Undecaprenyl-phosphate-galactosyltransferase is necessary for biosynthesis of O-antigen | NODE7_175907 | Frameshift | HIGH |
|  |  | Metabolism | *mtlA* | Membrane transport for mannitol | NODE4_129784 | Missense | MODERATE |
| **2** | PAST | Metabolism | *oadA* | Oxalacetate decarboxylase involved in the conversion of oxalacetate into pyruvate, alpha chain | NODE2_646874 | Missense | MODERATE |
|  | FUTURE | LPS byosinthesis | *rfbP* | Undecaprenyl-phosphate-galactosyltransferase is necessary for biosynthesis of O-antigen | NODE7_175907 | Frameshift | HIGH |
|  |  |  | *rfbD* | Rhamnosyltranferase involved in byosinthesis of O-antigen | NODE7_172620 | Stop gained | HIGH |
|  |  | Transcription | *23S* | 23s RNA its a component of large subunits 50S of ribosome | NODE22_405 | Intergenic | MODIFIER |
|  |  |  |  |  | NODE22_407 | Intergenic | MODIFIER |
|  |  |  |  |  | NODE22_408 | Intergenic | MODIFIER |
|  |  |  |  |  | NODE22_623 | Intergenic | MODIFIER |
|  |  | Phage related |  | Tail fiber component involved in recognition of receptors | NODE16_23549 | Missense | MODERATE |
|  |  |  | *mup48* | Mup48 protein | NODE16_21704 | Missense | MODERATE |

**Supplemental Table 1:** Complete SNP information for *S*. Enteritidis for the four independent replicates for day 1 (past) and day 21 (future). Specific genes with the mutation, the location into the genome, the effect type given by Freebayes, and the impact of the mutation.

| **Replicate** | **S. Enteritidis** | **Function** | ***Gen mutated*** | **Gene product activity** | **Chromosome position (bp)** | **Effect type** | **Putative impact** |
| --- | --- | --- | --- | --- | --- | --- | --- |
| **3** | PAST | Metabolism | *oadA* | Oxalacetate decarboxylase involved in the conversion of oxalacetate into pyruvate, alpha chain | NODE2_648483 | Missense | MODERATE |
|  |  |  |  |  | NODE2_648226 | Missense | MODERATE |
|  | FUTURE | Metabolism | *oadA* | Oxalacetate decarboxylase involved in the conversion of oxalacetate into pyruvate | NODE2_648837 | Frameshift | HIGH |
|  |  |  |  |  | NODE2_648794 | Missense | MODERATE |
|  |  |  |  |  | NODE2_648108 | Missense | MODERATE |
|  |  | Transcription | *23S* | 23S rNA, large subunit | NODE22_667 | Interegenic | MODIFIER |
|  |  |  |  |  | NODE22_671 | Interegenic | MODIFIER |
|  |  |  |  |  | NODE22_678 | Interegenic | MODIFIER |
|  |  |  |  |  | NODE22_679 | Interegenic | MODIFIER |
| **4** | PAST | Metabolism | *glnE* | Bifuntional enzyme involved in regulation of glutamine synthetase (GlnA) needed to assimilate ammonia | NODE2_499008 | Missense | MODERATE |
|  | FUTURE | LPS Byosinthesis | *rfbP* | Undecaprenyl-phosphate-galactosyltransferase is necessary for biosynthesis of O-antigen | NODE7_175907 | Frameshift | HIGH |
|  |  | Transcription | *23S* | 23S RNA its a component of large subunits 50S of ribosome | NODE22_630 | Intergenic | MODIFIER |
|  |  |  | *23S* | 23S RNA, small subunit | NODE23_1621 | Intergenic | MODIFIER |
|  |  | Phage related | *mup43* | Protein Mup43 is involved in cirularization of viral DNA | NODE16_18244 | Missense | MODERATE |
|  |  |  | *mup49* | Protein Mup49 Tail fiber component involved in recognition of receptors | NODE16_23549 | Missense | MODERATE |
|  |  | Secretion system III | *sscB* | Its a chaperone for SseF a effector protein involve in TSIII | NODE1_947512 | Missense | MODERATE |
|  |  | Unknown |  | Uncharacterized protein YggL | NODE2_402005 | Missense | MODERATE |
